# Draft Genome Sequence of *Microbacterium foliorum* Strain 122 Isolated from a Plant Growing in a Chronically Hydrocarbon-Contaminated Site

**DOI:** 10.1128/genomeA.00434-17

**Published:** 2017-05-25

**Authors:** Rhea Lumactud, Roberta Fulthorpe, Vladimir Sentchilo, Jan Roelof van der Meer

**Affiliations:** a Department of Physical and Environmental Sciences, University of Toronto Scarborough, Toronto, Ontario, Canada; b Department of Fundamental Microbiology, University of Lausanne, Lausanne, Switzerland

## Abstract

*Microbacterium foliorum* strain 122 is a bacterial endophyte isolated from a *Dactylis glomerata* plant growing in a natural oil seep soil located in Oil Springs, Ontario, Canada. We present here a draft genome sequence of an endophytic strain that has promising potential in hydrocarbon degradation and plant growth promotion.

## GENOME ANNOUNCEMENT

*Microbacterium foliorum* isolate 122 was isolated from a *Dactylis glomerata* plant growing in a chronically hydrocarbon-contaminated site located in Oil Springs, Ontario, Canada. This isolate was found to be common among all sampled plants. Using a colorimetric assay through the organisms' ability to reduce tetrazolium dye, this isolate was found to potentially respire petroleum hydrocarbon substrates, being strongly positive for octanol, toluene, naphthalene, kerosene, and motor oil, on the basis of purple color appearance (1). Using gas chromatography, *M. foliorum* 122 was able to mineralize 17% and 20% of toluene and naphthalene, respectively (1).

DNA was subjected to sequencing on a Pacific Biosciences RSII single-molecule real-time (SMRT) cell sequencing platform at the Lausanne Genomic Technologies Facility (University of Lausanne, Switzerland). A total of 77,004 reads were obtained, with an average length of 6,108 bp. The PacBio reads were assembled *de novo* using the SMRT Analysis suite and the HGAP 2.3 algorithm. The assembled contiguous chromosomal sequence was 3,743,121 bp, with a G+C content of 67.9%. Annotation was conducted using the RAST Web server (2, 3), generating 3,598 coding sequences, of which 43% were classified in 391 subsystems.

*M. foliorum* 122 contained 436 features associated with the subsystem carbohydrates, of which 98 were for monosaccharides, 80 were for disaccharides and oligosaccharides, 13 were for polysaccharides, 41 were for aminosugars, and 5 were for one-carbon metabolism, highlighting an endophytic lifestyle in relation to sugar metabolism. The pathways for tricarboxylic acid metabolism, glycolysis, and pyruvate metabolism were complete. The genome contained 3 features of a type II secretion system that is associated with pilus assembly essential for host colonization and adherence (4), as well as 76 features of ABC transporters. Genes for type I and III to VI secretion systems were absent. One prophage region was identified. *M. foliorum* 122 does not have genes for chemotaxis, but it contained 48 features related to flagellar motility. Despite the demonstrated utilization of toluene and naphthalene, no known genes for classical toluene, naphthalene, or biphenyl metabolism were found, but genes involved in catechol, salicylate, and benzoate degradation were detected. Genes for mercury and cobalt-zinc-cadmium resistance were found, and the presence of genes for multidrug resistance efflux pumps were identified. The genome also contained features involved in the biosynthesis of indole-3-acetic acid, an important hormone in plant growth and development.

This genome will be further tested with regard to its plant contaminant-degrading and plant growth-promoting capabilities, which may lead to biotechnological applications.

### Accession number(s).

This whole-genome sequencing project has been deposited at GenBank under accession no. CP019892. The version described in this paper is the first version, CP019892.
